# Scavenger Receptor Class B Member 1 Independent Uptake of Transthyretin by Cultured Hepatocytes Is Regulated by High Density Lipoprotein

**DOI:** 10.1155/2019/7317639

**Published:** 2019-06-18

**Authors:** Kelly A. Landers, Michael C. d'Emden, Kerry Richard

**Affiliations:** ^1^Conjoint Internal Medicine Laboratory, Chemical Pathology, Pathology Queensland, Queensland Health, Herston, QLD 4029, Australia; ^2^Department of Endocrinology and Diabetes, Royal Brisbane and Women's Hospital, Herston, QLD 4029, Australia; ^3^School of Medicine, University of Queensland, Herston, QLD 4029, Australia; ^4^School of Biomedical Sciences, Queensland University of Technology, Brisbane, QLD 4000, Australia

## Abstract

Thyroid hormone (thyroxine, T4) is essential for the normal function of all cell types and is carried in serum bound to several proteins including transthyretin. Recently, evidence has emerged of alternate pathways for hormone entry into cells that are dependent on hormone binding proteins. Transthyretin and transthyretin bound T4 are endocytosed by placental trophoblasts through the high-density lipoprotein receptor, Scavenger Receptor Class B Type 1 (SR-B1). High density lipoprotein (HDL) affects the expression and function of SR-B1 in trophoblast cells. SR-B1 is also expressed in hepatocytes and we sought to determine if hepatocyte SR-B1 was involved in transthyretin or transthyretin-T4 uptake and whether uptake was affected by HDL. Transthyretin and transthyretin-T4 uptake by hepatocytes is not dependent on SR-B1. HDL treatment reduced SR-B1 expression. However, pretreatment of hepatocytes with HDL increased uptake of transthyretin-T4. Knockdown of SR-B1 expression using siRNA also increased transthyretin-T4 uptake. Coaddition of HDL to transthyretin uptake experiments blocked both transthyretin and transthyretin-T4 uptake. Hepatocyte uptake of transthyretin-T4 uptake is influenced by, but is not dependent on, SR-B1 expression. HDL also decreases transthyretin-T4 uptake and therefore diet or drugs may interfere with this process. This suggests that multiple lipoprotein receptors may be involved in the regulation of uptake of transthyretin-T4 in a cell-type specific manner. Further study is required to understand this important process.

## 1. Introduction

Transfer of thyroid hormone into cells is critical for normal physiology. Free thyroid hormone is known to enter cells through specific cell surface transport proteins, including monocarboxylate transporters 8 and 10, and organic anion transporting polypeptides [1]. For many years this uptake of unbound thyroid hormone was assumed to be the only relevant mechanism, supporting the free hormone hypothesis [2].

The thyroid hormone binding protein transthyretin (TTR) is also produced by and endocytosed by many different cell types [3–5]. Liver is the major site of production and secretion of serum TTR and is also its main site of catabolism [6]. TTR is the major protein found in human cerebrospinal fluid (25% of total protein) and is synthesized and secreted into CSF by choroid plexus epithelial cells [7]. TTR is then endocytosed by Megalin (LRP2)[4], an endocytic multiligand receptor of the low-density lipoprotein (LDL) receptor family, in brain astrocytes [8, 9] with uptake increased in the presence of thyroid hormone in the form of thyroxine (T4). TTR synthesis by choroid plexus is essential for distribution of T4 around the brain [10]. TTR uptake by kidney is also mediated by Megalin expressed in the epithelium of renal proximal tubules. TTR uptake by liver is also thought to be* via* a lipoprotein receptor [5]; however the specific receptor has not yet been identified. TTR is a carrier of thyroid hormone; it transports retinol through its association with retinol binding protein; it binds many endo- and xenobiotics (in particular endocrine disrupting chemicals); it is involved in the regulation of astrocytic glycolysis [11]; and mutations in TTR lead to hereditary amyloidosis [12].

TTR is also synthesized by human placenta [13] and its endocytosis by trophoblasts [3] is enhanced by the presence of T4 [3]. This receptor mediated uptake of transthyretin bound to thyroxine (TTR-T4) into placental trophoblasts is dependent on expression of Scavenger Receptor Class B Type 1 (SR-B1) which is also a high-density lipoprotein (HDL) receptor [14]. TTR-T4 uptake by trophoblasts was increased by pretreatment of cells with HDL for four hours, and HDL also competes with TTR for uptake through SR-B1 [14].

Given the importance of T4 for liver cell function, the importance of liver in both TTR and T4 metabolism, and strong expression of SR-B1 in hepatocytes [15], we sought to determine if SR-B1 and/or HDL are important in the regulation of hepatocyte TTR and TTR-T4 uptake.

## 2. Methods

### 2.1. Reagents

Transthyretin (Prealbumin from human serum) was purchased as a lyophilised powder from Sigma Aldrich, Victoria, Australia. High-density lipoprotein (HDL) from human plasma was purchased as a solution from Sigma Aldrich, Victoria, Australia. Unless otherwise stated reagents were from Sigma Aldrich, Victoria, Australia.

### 2.2. Cell Culture

The human hepatocyte cell line, HepG2 (HB-8065), was obtained from the American Type Culture Collection (ATCC) (VA, USA) and maintained in Dulbecco's Modified Eagle's Medium (DMEM) (Sigma, Castle Hill, NSW, Australia) /10% FBS in a humidified atmosphere of 95% air and 5% CO_2_.

### 2.3. Knockdown of SR-B1 Expression Using Mission esiRNA

Knockdown of SR-B1 was achieved using MISSION esiRNA (Sigma) in combination with Lipofectamine RNAiMAX transfection reagent (Thermo Fisher) as per manufacturer's instructions and as previously described [14]. Kif11a esiRNA was used as a positive transfection control and EGFP esiRNA as a mock control. Cells were cultured in 24-well plates (50 000 cells/well) and grown overnight. After 24 hours, culture medium was replaced with antibiotic free medium. Subsequently, 1.2 *μ*M MISSION esiRNA in Opti-MEM and 0.04% (V/V) Lipofectamine RNAiMAX in Opti-MEM were incubated together before adding to cells for 24 hours. This transfection process was repeated after 24 hours. Cells were used in experiments 24 hours after the final transfection.

### 2.4. Alexa–TTR Labelling

Purified native human TTR protein was labelled using an Alexa Fluor® 568 Protein Labelling Kit (Life Technologies, VIC, Australia). Briefly, 1 ml of 2 mg/ml purified TTR protein was incubated with Alexa Fluor® 568 reactive dye for 2 hours at room temperature. Then Alexa Fluor 568 labelled-TTR (Alexa-TTR) was purified through a Bio-Gel P-6 Gel column (BioRad, CA, USA). To determine the concentration of labelled TTR and the degree of fluorescent labelling, the conjugate solution was measured at 280 nm and 540 nm in a NanoDrop 2000 spectrophotometer (Thermo Scientific, NC, USA).

### 2.5. Quantification of Alexa568-TTR Uptake

HepG2 cells (~50,000 cells/well) were cultured on 24-well plates for 48-72 hours. Following serum starvation for 4 hours, cells were incubated with 1uM Alexa568-TTR ± 10*μ*M T4 as previously published [3] and imaged using an IncuCyte ZOOM™ live cell-imaging system (Essen BioScience, MI, USA) with Phase and Red channels and scanned at 2-3 hours' intervals for up to 48 hours. IncuCyte ZOOM software was used to quantify the red objects in 9 nonoverlapping images per well (the average of these 9 values is equivalent to n=1). Data were presented as mean number of red objects per image relative to percentage confluence.

### 2.6. Western Blotting

Twenty *μ*g of total cell lysate was run on NuPAGE Novex Bis-Tris gradient gels (Invitrogen). Proteins were transferred to nitrocellulose and probed using the following antibodies from Sigma: rabbit anti-SR-B1, 1:750, and mouse anti-*β*-actin, 1:2000. Blots were developed using a SuperSignal West Femto Kit (Pierce, Scoresby, VIC, Australia) and imaged using a LAS-4000 (Fujifilm, Brookvale, NSW, Australia). Blots were quantified using ImageJ software (NIH).

### 2.7. Statistical Analysis

Statistical analysis was performed using Microsoft Excel or GraphPad Prism Software (GraphPad Software, Inc., CA, USA). Comparison between groups was determined by one-way ANOVA with Tukey's test or by paired Student's t-test. P values of ≤0.05 were considered significant.

## 3. Results

### 3.1. Effect of HDL Treatment on SR-B1 Levels in HepG2 Cells

**Figure 1:**
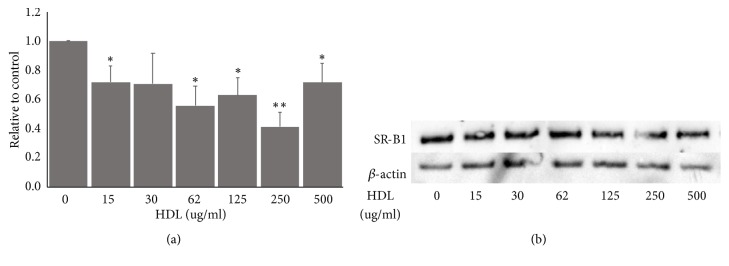
*Treatment with high density lipoprotein (HDL) reduces scavenger receptor class B type 1 (SR-B1) expression in HepG2 cells.* (a) Treatment with 15, 62, 125, 250, and 500 ug/ml HDL all significantly reduced protein levels of SR-B1. (b) Representative Western blot showing reduction of SR-B1 levels. Cells were treated with 15–500 ug/ml HDL for 24 hours. Cell lysates were collected and run on 4-12% Bis-Tris gels, transferred to nitrocellulose, probed with anti-SRB1 (1:750) and anti-*β*-Actin (1:2000) antibodies, and developed using enzyme linked chemiluminescence. Protein levels were quantitated using ImageJ software (*∗∗* p< 0.01, *∗*p<0.05; 15-125 ug/ml, n=4 and 250-500 ug/ml, n=2).

SR-B1 is an HDL receptor and incubation with HDL increases SR-B1 levels in trophoblasts [14]. The normal reference range for serum HDL is 400 to 600 ug/ml and individuals with HDL values less than 400 ug/ml are at increased risk of heart disease. We tested whether HDL (0–500 ug/ml) influenced SR-B1 levels in hepatocyte cells. Following four hours in serum free medium, treatment with HDL for 24 hours resulted in significantly decreased SR-B1 levels but the effect was not dose dependent. 250 ug/ml HDL reduced SR-B1 levels to less than 50% of control (p =0.001, n=2) and 15 ug/ml HDL reduced SR-B1 levels to 70% of control (p=0.04, n=4) (Figure 1).

### 3.2. Uptake of Alexa-TTR in HDL Treated Cells

HDL decreases SR-B1 expression in HepG2 cells but HDL is also transported through SR-B1 and may compete with TTR and TTR±T4 uptake through SR-B1 when both HDL and TTR are present together in the uptake medium [14]. We therefore also tested whether coincubation with HDL influences TTR (± T4) uptake through SR-B1 in HepG2 cells. We preincubated with either no HDL (untreated) or 500 ug/ml HDL (pretreated /normal serum level of HDL) and then coincubated with 500 ug/ml (normal serum level) or 1000 ug/ml (high serum level) of HDL.

#### 3.2.1. Pretreatment of Cells with HDL

**Figure 2:**
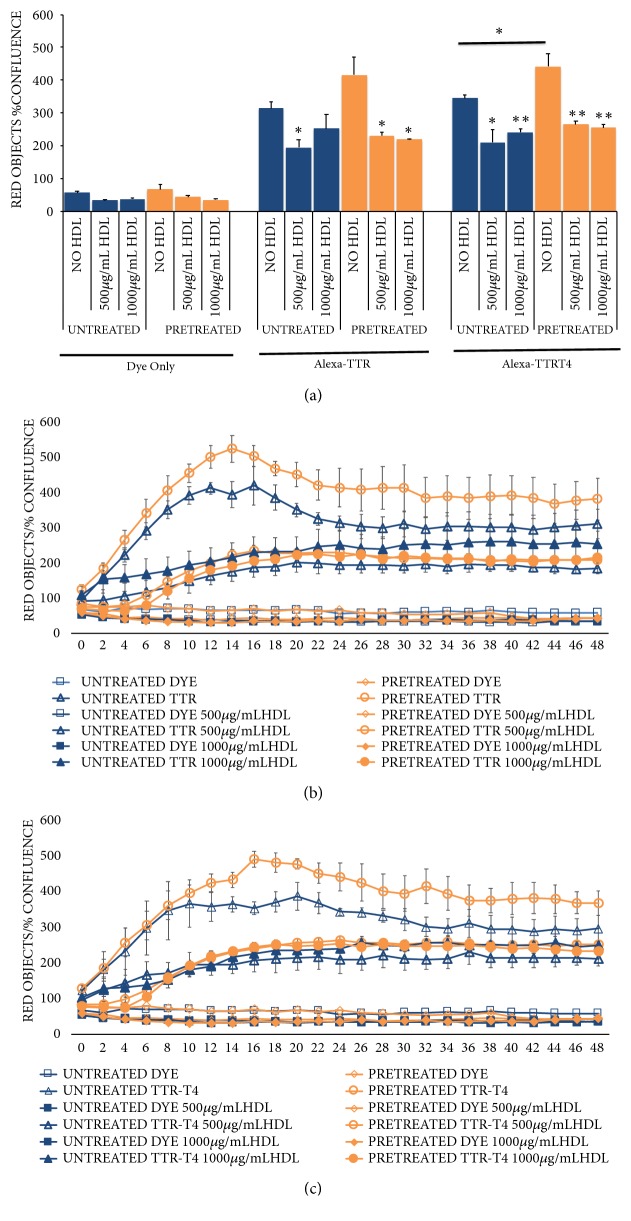
*The effects of high density lipoprotein (HDL) treatment on TTR and TTR-T4 uptake in HepG2 cells.* Cells were serum starved for 4 hours before uptake experiments. 500 ug/ml HDL was added to the serum free medium during this 4-hour period for the HDL ‘pretreated' group. Medium was removed and replaced with serum free medium containing unconjugated Alexa dye, Alexa-labelled TTR, or Alexa-labelled TTR-T4 (± 500/1000 ug/ml HDL) and cells were transferred to an Essen IncuCyte incubator where intracellular fluorescence and cell confluence (%) were measured every 2 hours. Alexa-TTR uptake is expressed as red objects/% confluence. (a) Relative uptake of dye, Alexa-TTR, and Alexa-TTR-T4 at 24 hours. Pretreatment with 500 ug/ml HDL for 4 hours resulted in greater uptake of Alexa-TTR-T4 at 24 hours (p<0.05, n=4). Cotreatment with 500 ug/ml HDL resulted in reduced uptake of Alexa-TTR and Alexa-TTR-T4 in all conditions tested. Coincubation with 1000 ug/ml HDL reduced uptake of Alexa-TTR in cells pretreated with HDL only. 1000 ug/ml HDL reduced uptake of Alexa-TTR-T4 in both control and pretreated cells (n=4, *∗*p<0.05, *∗∗*p<0.01). (b) Uptake of Alexa-TTR over 48 hours. Coincubation with HDL reduced Alexa-TTR uptake under all conditions (n=4). (c) Uptake of Alexa-TTR-T4 over 48 hours. Coincubation reduced Alexa-TTR-T4 uptake under all conditions (n=4).

Pretreatment of HepG2 cells with 500 ug/ml HDL in serum free medium for 4 hours (orange bars) resulted in no significant change in uptake of Alexa-TTR at 24 hours compared to control (blue bars) (Figures 2(a) and 2(b)). Pretreatment with HDL resulted in increased uptake of Alexa-TTR-T4 at 24 hours (441.1±39.5 vs. control 344.5±9.3, n= 4, p <0.05) (Figures 2(a) and 2(c)). Despite pretreatment with HDL leading to increased uptake of Alexa-TTR-T4 at 24 hours, we were unable to demonstrate any significant changes in SR-B1 protein levels following 4 hours' pretreatment in 500ug/ml HDL (data not shown).

#### 3.2.2. Coaddition of HDL into the Alexa-TTR Uptake Medium Results in Decreased Uptake of Alexa-TTR and Alexa-TTR-T4

At 24 hours, coincubation with 500 ug/ml HDL resulted in significantly reduced uptake of Alexa-TTR in both untreated (blue bars, 194.5±23.8, n=4, p=0.01) and HDL pretreated (orange bars, 230.3±11.8, p<0.05) cells when compared to controls (313.3±20.8 and 415.0±54.7, resp., n=4). 1000 ug/ml HDL resulted in significantly decreased uptake of Alexa-TTR in HDL pretreated cells only( 219.7±1.8, n=4, p<0.05) (Figure 2).

At 24 hours, coincubation with 500 ug/ml HDL or 1000 ug/ml HDL resulted in significantly reduced uptake of Alexa-TTR-T4 in both untreated and HDL pretreated cells. In untreated cells, 500 ug/ml HDL reduced Alexa-TTR-T4 uptake to 210.1±38.4 (n=4, p= 0.01) and 1000 ug/ml HDL reduced uptake to 240±12.4 (n=4, p=0.001) when compared to control cells (344.5±9.3, n=4). In cells pre-reated with 500 ug/ml HDL for 4 hours and then washed before uptake experiments, 500 ug/ml reduced Alexa-TTR-T4 uptake to 265.2±9.5 (n=4, p=0.005) and 1000 ug/ml HDL reduced uptake to 254.4±10.0 (n=4, p<0.005) when compared to control cells (441.1±39.5, n=4) (Figure 2).

Coincubation with HDL resulted in decreased uptake of TTR (Figure 2(b)) and TTR-T4 (Figure 2(c)) consistently across a 48-hour experiment.

### 3.3. Expression of SR-B1 in Hepatocyte Cell Lines and Knockdown of SR-B1

**Figure 3:**
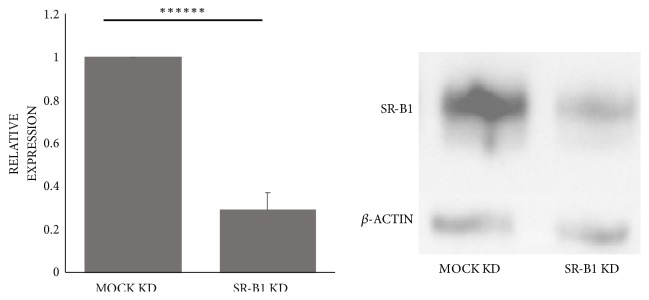
*Scavenger receptor Class B Type 1 expression in HepG2 cells.* Using esiRNA, SR-B1 levels were knocked down to 29±8% of mock knockdown (KD) control levels (n=4, *∗∗∗∗∗∗*p<0.00001).

SR-B1 is strongly expressed by HepG2 cells [16]. Using esiRNA, SR-B1 protein was consistently knocked down to 29±8% (n=4, p=2.0 x 10^−7^) of mock control in HepG2 cells (Figure 3).

### 3.4. Effect of SR-B1 Knockdown on Alexa-TTR (± T4) Uptake in HepG2 Cells

**Figure 4:**
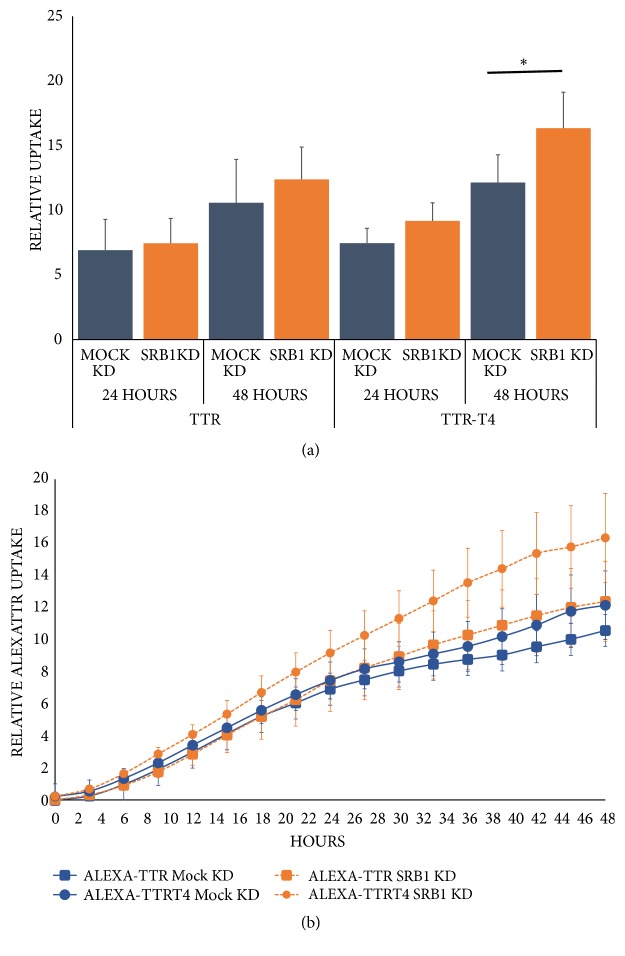
*Scavenger receptor Class B Type 1 (SR-B1) levels affect uptake of Alexa-TTR-T4 by HepG2 cells. *(a) Knockdown of SR-B1 levels (to 29±8% of mock control levels) prior to uptake experiments resulted in increased (~133%) uptake of Alexa-TTR-T4 at 48 hours compared to control. (b) Comparison of Alexa-TTR (square) uptake in mock control (blue) versus SR-B1 knockdown (orange) cells and of AlexaTTR-T4 (circle) uptake in mock control versus SR-B1 knockdown cells (all n=4, *∗*p<0.05).

We then tested the effect of SR-B1 knockdown in HepG2 cells on TTR (± T4) uptake. SR-B1 knockdown resulted in significantly increased uptake of TTR-T4 only at 48 hours (to ~133% of uptake of mock control cells, Figure 4, n=4, p<0.05).

## 4. Discussion

This study has demonstrated that HepG2 cells take up TTR and the TTR-T4 complex. TTR is more rapidly taken up by cells when it is bound to T4 [3, 5, 8]. T4 binding stabilises the TTR homotetramer and it is this conformation that is more readily recognised by membrane receptors [3]. Pretreatment of HepG2 cells with HDL for 4 hours results in increased uptake of Alexa-TTR-T4 (although no changes in protein level were detected following 4 hours in HDL). However, coincubation of cells with HDL and Alexa-TTR (or Alexa-TTR-T4) results in reduced uptake of both Alexa-TTR and Alexa-TTR-T4. This suggests that HDL competes with and blocks TTR and TTR-T4 uptake by hepatocytes. Around 1-2% of TTR circulates in serum bound to lipoproteins [17] and HDL has previously been shown to affect uptake of unbound T4 into human fibroblasts [18] and uptake of TTR and TTR-T4 by placental trophoblasts [14].

Knocking down SR-B1 expression has no significant effect on uptake of Alexa-TTR but surprisingly increases uptake of Alexa-TTR-T4. These results suggest that TTR-T4 uptake is not dependent upon SR-B1 expression but is inversely influenced by SR-B1 expression levels. A previous study also speculated that SR-B1 may be involved in TTR uptake and attempted to compare uptake in two CHO cell lines but were unable to show any differences in uptake, although the data was not presented in the publication [5]. However, in agreement with our study, the study did demonstrate that HDL was able to reduce TTR uptake in rat hepatoma cells [5]. Sousa* et al.* also showed that lipoproteins inhibited TTR internalisation; however they also demonstrated that uptake was not lipoprotein dependent or due to TTR-lipoprotein complexes [5]. Our current study unequivocally demonstrates that, unlike trophoblasts [14], SR-B1 is not the receptor responsible for TTR uptake in hepatocytes; however TTR-T4 uptake is at least influenced by SR-B1 levels.

Aside from TTR, T4 is carried in serum by thyroxine binding globulin (TBG) and albumin. TTR-T4 uptake has previously been described in placenta [3], astrocytes [9], choroid plexus [7], retinal pigment epithelial cells [19, 20], and kidney [4, 5]. TBG has a strong binding affinity for T4; however uptake of TBG-bound T4 has not been previously described. Albumin has a weak binding affinity for T4 and although cellular uptake of albumin is common; there is also no current evidence of uptake of albumin-T4 by cells.

Megalin is a membrane receptor that has been shown to be involved in TTR uptake by other tissues such as kidney [4] and astrocytes [9]. Megalin is not expressed by liver and specifically not in HepG2 [21] cells. Aside from SR-B1 and Megalin, no other TTR receptors have been identified to date.

The hepatocyte lipoprotein receptor involved in TTR and TTR-T4 uptake remains elusive. It may be that this process is so fundamental to TTR metabolism that the uptake mechanism is redundant and several lipoprotein transporters are capable of carrying out this important role making it difficult to identify one specifically.

The TTR uptake mechanism may be particularly sensitive to changes in local TTR levels (total of serum TTR and locally secreted TTR) making it important in cells that both secrete and endocytose TTR (e.g., placental trophoblast [3, 13] and hepatocytes [5]) or cells in close proximity to cells that secrete TTR (e.g., neurons/astrocytes [11]).

Coincubation with HDL decreases TTR and TTR-T4 uptake and it therefore seems possible that TTR turnover (synthesis, secretion, uptake, and degradation) is affected by diet and nutrition. The liver is very sensitive to reduced dietary protein intake and shuts down protein synthesis very rapidly. Since TTR has a very short half-life of only 2–4 days [22], serum levels of TTR are routinely used as a marker of malnutrition and have appeared since the 1970s [23]. Additionally, a reduction in serum levels of lipoproteins should lead to increased uptake of TTR by hepatocytes, leading to increased intracellular degradation. Therefore, a starvation diet/malnutrition would likely result in reduction of serum TTR levels due to both reduced TTR synthesis and increased TTR uptake and catabolism. Likewise, diet or drugs that interfere with cholesterol metabolism (such as statins [24]) may also affect TTR turnover. Any increase or decrease in circulating levels of lipoproteins would likely have an effect on TTR uptake and metabolism.

## 5. Conclusion

The data from this study concludes that TTR-T4 uptake by cultured hepatocytes is not dependent on SR-B1 expression but TTR-T4 uptake is inversely influenced by SR-B1 expression levels. Since HDL levels influence TTR-T4 uptake by liver, clearly the effect of drugs or a diet that contributes to changes in serum levels of TTR and lipoproteins will have an effect on hepatocyte TTR (and TTR-T4) uptake and subsequent turnover of TTR. Further investigation of the mechanisms of liver TTR- transport will shed more light on this important process.

## Data Availability

The data used to support the findings of this study are available from the corresponding author upon request.
